# The Role of Hedgehog Signalling in the Formation of the Ventricular Septum

**DOI:** 10.3390/jdb5040017

**Published:** 2017-12-12

**Authors:** Antonia Wiegering, Ulrich Rüther, Christoph Gerhardt

**Affiliations:** Institute for Animal Developmental and Molecular Biology, Heinrich-Heine University Düsseldorf, 40225 Düsseldorf, Germany; antonia.wiegering@hhu.de (A.W.); ruether@hhu.de (U.R.)

**Keywords:** ventricular septal defect, VSD, Smoothened, SMO, SAG, purmorphamine, oxysterols, GSA-10, cilia, Down syndrome

## Abstract

An incomplete septation of the ventricles in the vertebrate heart that disturbes the strict separation between the contents of the two ventricles is termed a ventricular septal defect (VSD). Together with bicuspid aortic valves, it is the most frequent congenital heart disease in humans. Until now, life-threatening VSDs are usually treated surgically. To avoid surgery and to develop an alternative therapy (e.g., a small molecule therapy), it is necessary to understand the molecular mechanisms underlying ventricular septum (VS) development. Consequently, various studies focus on the investigation of signalling pathways, which play essential roles in the formation of the VS. In the past decade, several reports found evidence for an involvement of Hedgehog (HH) signalling in VS development. In this review article, we will summarise the current knowledge about the association between HH signalling and VS formation and discuss the use of such knowledge to design treatment strategies against the development of VSDs.

## 1. Introduction

**Figure 1:**
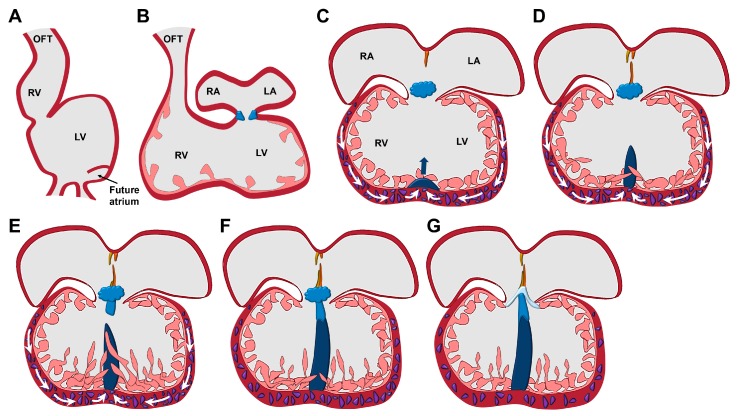
Development of the ventricular septum. (**A**) The linear heart tube balloons to give rise to precursor structures of the heart chambers. (**B**) The heart takes its four-chambered shape by a process termed heart looping. (**C**) Proliferating cells (in purple) of the ventricular walls lead to the outgrowth of the muscular ventricular septum (in dark blue). (**D**) In addition, trabeculae that are derived from the ventricular walls start to participate in the formation of the ventricular septum. (**E**) After a molecular interaction between the muscular ventricular septum and the endocardial cushion cells (in bright blue), the membranous ventricular septum develops from the endocardial cushion cells and grows towards the muscular ventricular septum. (**F**) Finally, the muscular and membranous ventricular septa fuse. (**G**) The atrioventricular endocardial cushion cells give rise to the atrioventricular valves. LA, left atrium; LV, left ventricle; OFT, outflow tract; RA, right atrium; RV, right ventricle.

100 years ago, David Waterston reported that the ventricular septum (VS) grows out from the anterior bulboventricular groove [1]. In 1940, John Ernest Frazer identified the atrioventricular endocardial cushion cells as the original source of VS formation [2], causing a controversial debate about the place of origin from which the ventricular septum is formed. In the 1970s, investigations on congenital heart diseases revealed that the ventricular septum consists of different structures that are built up from different starting points [3,4,5,6]. The formation of the muscular part of the ventricular septum starts concomitantly with the ballooning of the linear heart tube [7]. During this ballooning, the heart chambers are specified and the bulboventricular groove develops [8] (Figure 1A–C). From the bulboventricular groove, the muscular ventricular septum arises (Figure 1C–E), while the membranous part of the ventricular septum is formed by the atrioventricular endocardial cushion cells [9] (Figure 1E,F). The development of the muscular ventricular septum is based on the proliferation of cells that are localized in the ventricular walls and takes place concomitantly with the growth of the both ventricles [10,11] (Figure 1C–E). Moreover, many of the trabeculae (intraventricular collections of linearly ordered myocytes) fuse with the growing muscular ventricular septum and accelerate its outgrowth (Figure 1D,E) [12,13]. Since the trabeculae are derived from the myocardial cells of the ventricular walls [14,15,16], the proliferation of the ventricular wall cells is of outstanding importance for the formation of the muscular ventricular septum [10,17,18]. Finally, the muscular VS comprises a mixture of cardiomyocytes derived from the left and the right ventricle [11]. The muscular VS, after its formation, interacts with the atrioventricular endocardial cushion cells in a yet unknown manner, thereby initiating the development of the membranous VS [9,19,20]. The membranous VS grows from the atrioventricular endocardial cushion cells towards the muscular VS until they finally fuse together [19,21,22] (Figure 1E–G).

An incomplete septation of the ventricles is known as a ventricular septal defect (VSD) and allows for the communication of the left and the right ventricle, which, in turn, results in the mixture of oxygenated and de-oxygenated blood and an increase of the blood flow towards the lung and the left ventricle [23]. The consequence is the emergence of pulmonary edema and dilatation, as well as hypertrophy of the left ventricle [24,25]. Furthermore, the blood flow towards other organs in the body is impaired, and hence their supply of oxygen and nutrients is not ensured. The severity of the defect is dependent on the size of the VSD. The first clinical description of a VSD was provided in 1879 [26]. Together with bicuspid aortic valves, the VSD is the most common congenital heart disease in humans today [27,28,29]. In most cases, children do not die due to VSDs, but they often suffer from defective heart function as adults [23,30]. To prevent these severe consequences, VSDs are currently treated with surgery. Although surgical repair is the most frequently performed procedure in pediatric cardiac surgery, and although this treatment is successfully applied in most cases [31,32], surgical closure of VSDs entails several risks, like chronotropic incompetence, operative mortality, or late death [32,33]. To avoid surgery and to develop alternative therapies to treat VSDs, it is necessary to know the molecular mechanisms underlying ventricular septal development. Since these mechanisms are largely unknown [11,20], a recent report attracted widespread attention. Li et al. reported that a plethora of congenital heart defects in mice are caused by mutations in genes encoding ciliary proteins [34]. Ciliary proteins are essential for the function of cilia, little hair-like, cellular protrusions that can be subdivided into two groups. Simplified, one differentiates between motile cilia and immotile cilia [35]. Immotile cilia are also known as primary cilia. They receive signals from their environment, mediate them, and transduce these signals into the cell’s interior. Due to this signal transduction, certain gene expressions are regulated within the cell nucleus, and, in turn, cellular processes like proliferation, apoptosis, and differentiation are initiated [36,37]. A signalling pathway that depends highly on the mediation by primary cilia and plays an important role in the development of numerous vertebrate organs is the Hedgehog (HH) signalling pathway [38,39,40,41,42,43,44].

**Figure 2:**
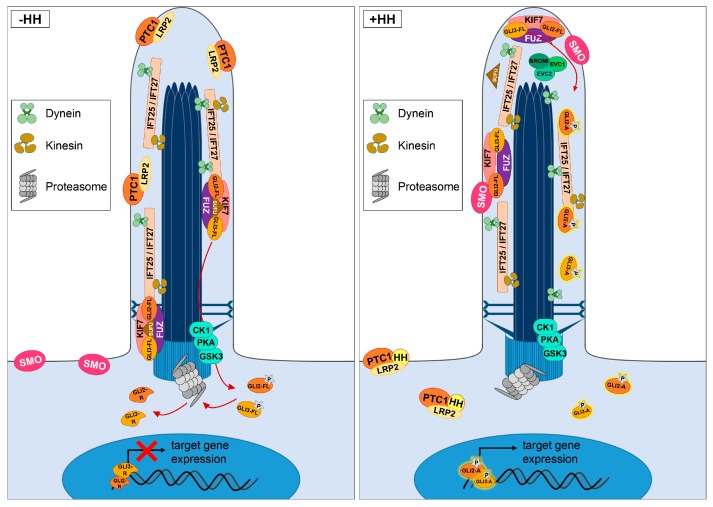
Hedgehog signalling at primary cilia.

In vertebrates, HH signalling starts with the binding of the ligand HH [three different vertebrate HH proteins exist—Sonic hedgehog (SHH), Indian hedgehog (IHH), and Desert hedgehog (DHH)] to its receptor Patched (PTC1), which is located in the membrane of primary cilia (Figure 2). One factor that promotes this binding event is Low-density lipoprotein receptor related protein 2 (LRP2) [45]. After the binding event, the HH/PTC1 complex exits the cilium and Smoothened (SMO) is allowed to enter the ciliary membrane [46,47]. Upon arriving in the ciliary membrane, SMO dissociates the full-length Glioblastoma 2 (GLI2) and Glioblastoma 3 (GLI3) proteins from Suppressor of Fused (SUFU) via a yet unknown mechanism and transforms them into transcriptional activators (GLI2-A and GLI3-A) [48,49]. This transformation is enabled by the action of the protein Broad-Minded (BROMI; also referred to as TBC1D32) and the ciliary proteins Ellis Van Creveld 1 (EVC1) and Ellis Van Creveld 2 (EVC2) [50,51,52,53,54,55,56]. Subsequent to their activation, GLI2-A and GLI3-A translocate into the nucleus and induce HH target gene expression (e.g., the expression of *Gli1* or *Ptc1*). The transport of PTC1, SMO, and GLI2 within primary cilia was shown to be dependent on the Intraflagellar transport proteins 25 and 27 (IFT25 and IFT27) [57,58,59]. Loss of either IFT25 or IFT27 leads to reduced HH target gene expression [57,58]. In the absence of HH, PTC1 stays within the ciliary membrane and SMO remains outside the cilium. As a consequence, the cilia-regulated proteasome proteolytically processes the full-length GLI2 and GLI3 proteins into transcriptional repressors (GLI2-R and GLI3-R) [60,61,62]. This proteolytic processing event starts with the phosphorylation of GLI2 and GLI3 by protein kinase A (PKA), Casein kinase 1 (CK1), and Glycogen synthase kinase 3-β (GSK3-β) [63,64,65]. Furthermore, the proteins Kinesin family member 7 (KIF7) and Fuzzy (FUZ) seem to be involved in the processing of GLI2 and GLI3, and also in the conversion of their full-length forms into transcriptional activators by yet unknown mechanisms [66,67,68,69,70].

In vertebrates, HH signalling is mediated by primary cilia. The transport of proteins through the cilium is performed by the motor proteins Kinesin and Dynein, as well as by IFT proteins, in the case of HH signalling components especially by IFT25 and IFT27. In the absence of the HH ligand, PTC1 prevents the ciliary entry of SMO. GLI2-FL and GLI3-FL are bound to SUFU and get phosphorylated by PKA, CK1, and GSK3-β. Subsequently, GLI2-FL and GLI3-FL undergo proteolytic processing, which is realised by the cilia-regulated proteasome. Moreover, KIF7 and FUZ were reported to be involved in the proteolytic processing of these proteins. The products of this processing event, GLI2-R and GLI3-R, translocate into the nucleus to inhibit HH target gene expression. In the presence of HH, the ligand binds to PTC1. This binding is supported by the co-receptor LRP2. After this binding event, the PTC1/LRP2/HH complex exits the cilium allowing SMO to enter the cilium. Within the cilium, SMO induces the dissociation of the GLI2-FL and GLI3-FL proteins from SUFU. With the support of BROMI, EVC1, and EVC2, SMO modifies GLI2-FL and GLI3-FL into the transcriptional activators GLI2-A and GLI3-A (most likely by phosphorylation). After this modification, GLI2-A and GLI3-A activate HH target gene expression.

## 2. HH Signalling Plays an Essential Role in the Development of the VS

To evaluate the importance of HH signalling in the formation of the human VS, cardiac investigations of mice in which HH signalling components are truncated or inactivated give expressive statements (for an overview see Table 1). *Shh*^−/−^ mice are not viable and *Shh*^−/−^ mouse embryos display several heart defects, including, inter alia, atrioventricular septal defects (AVSDs) [71]. In contrast to *Shh* mutant mice, until now, congenital heart defects were not described in *Ihh* or *Dhh* mutant mice. Mutation of *Lrp2* results in the development of persistent truncus arteriosus, aortic arch defects, and VSDs in murine embryos [34]. *Sufu* mutant mouse embryos display ventricular septal defects and other cardiac defects, while *Bromi* mutant mouse embryos exhibit, amongst other heart malformations, AVSDs [34]. Moreover, *Ift25*^−/−^ and *Ift27*^−/−^ mouse embryos display VSDs, AVSDs, and other heart defects [57,58]. Consequently, mutations in many genes whose products positively control HH signalling result in the development of VSDs in mice, indicating that HH signalling is important for proper VS development. However, mutations in several genes that encode negative regulators of HH signalling also lead to the occurrence of VSDs. In this context, the loss of GSK3-β in mice results in the development of VSDs [72]. Furthermore, *Kif7* and *Fuz* mutant mouse embryos show AVSDs [34,73]. These three proteins, GSK3-β, KIF7, and FUZ, are necessary for proteolytic processing of GLI2 and GLI3, indicating that the inhibition of HH target gene expression might be an important factor for proper VS development. If this hypothesis is true, lack of either GLI2-R or GLI3-R should lead to the onset of VSDs. When considering that GLI3-R is the main transcriptional repressor in the HH pathway, the VS formation in *Gli3*^−/−^ mouse embryos is the best parameter to test this hypothesis. Remarkably, GLI3 deficiency does not result in the development of VSDs [74]. This finding is a very good hint for the possibility that the downregulation of HH signalling has a negative rather than a positive effect on the outgrowth of the VS in mice. Assuming that the GLI3-R is not a decisive factor in VS formation, it is conceivable that the occurrence of VSDs in *Gsk3-β*^−/−^, *Kif7*^−/−^ and *Fuz*^−/−^ mouse embryos results from the influence of these genes and their products in other signalling pathways than the HH pathway. It was previously reported that GSK3-β participates in the regulation of several signalling pathways, like canonical WNT signalling, NOTCH signalling and TGF-β signalling which participate in the formation of the VS [75,76,77,78,79,80,81,82,83]. KIF7 and FUZ have an effect on other signalling pathways via their involvement in ensuring proper cilia function [66,69,70,84,85,86,87,88]. Moreover, KIF7 and FUZ do not only take part in the generation of the GLI3-R, but also in the production of the GLI3-A [66,67,68,69,70], making it possible that VSDs in *Kif7*^−/−^ and *Fuz*^−/−^ mouse embryos are caused by a reduced amount of the GLI3-A. Importantly, the significant role of HH signalling in the development of the VS is conserved in humans. Patients carrying mutations in EVC1 or EVC2 suffer from VSDs and other cardiac malformations [89,90]. Furthermore, an association between a reduced expression of the HH target gene GLI1 and the occurrence of VSDs was reported in Down syndrome patients [91]. In summary, these data obtained from studies in patients and mouse models indicate that HH signalling is essential for proper VS development and that any disruption of this pathway can lead to the development of VSDs.

HH signalling regulates VS formation at different time points and from various locations. In mice, the first influence of HH signalling on the development of the VS seems to be at around embryonic day (E) 7.0 to E7.5. At this time, HH signalling from the pharyngeal endoderm ensures the proper genesis of the second heart field [71,92,93,94,95,96,97]. It governs the proliferation of second heart field cells, which are cardiac progenitor cells in the pharyngeal mesoderm, via the Wnt/β-catenin signalling pathway, the T-box transcription factor 5 (TBX5), and Forkhead box transcription factors 1a and 2 (FOXF1a and FOXF2) [78,98,99,100]. These cells attach to the growing arterial and venous poles of the linear heart tube in order to drive its elongation [94,101]. Subsequent to this elongation event, the linear heart tube is formed into a four-chambered heart by two processes, which are termed heart looping and septation. The heart looping process depends on the establishment of the left-right asymmetry that is realised at the node (a unique cohort of cells at the anterior tip of the primitive streak during gastrulation) in mammals [102]. In mice, the establishment of left-right asymmetry takes place at E7.75 to E8.0 [103]. At this time, HH is essential for the proper establishment of left-right asymmetry at the node. So called nodal vesicular parcels (NVPs), which contain HH, are transported to the left side of the node via the nodal flow that is created by the movement of motile cilia [104]. Upon arrival at the left side of the node (lateral plate mesoderm), HH initiates a signalling cascade that determines left identities in mice [105]. Remarkably, HH signalling controls the establishment of left-right asymmetry not only in mice, but also in other vertebrates [106,107,108,109]. If the establishment of left-right asymmetry fails, the organs within the vertebrate body are distributed randomly (heterotaxy) [110]. As one consequence of this defect, VSDs can occur since impaired left-right asymmetry affects the looping of the linear heart tube. In turn, this disturbed looping causes a disrupted arrangement of atrioventricular endocardial cushion cells, which are essential for proper membranous VS formation [111]. Furthermore, second heart field defects often result in VSDs [78,93,98,112,113,114]. It was previously reported that second heart field defects can result in an impaired development of the outflow tract [115,116,117]. Since it is known that the development of the muscular VS can be affected by outflow tract defects [118], it is conceivable that the occurrence of these outflow tract defects finally provoke the development of VSDs under second heart field defect conditions. Thus, HH signalling controls the development of the VS from two different regions outside the heart.

In addition to extracardiac HH signalling, intracardiac HH signalling participates in the formation of the VS. Cilia-mediated HH signalling governs the proliferation of myocardial cells in distinct regions of the ventricular walls [119]. The decrease of HH signalling due to a dysfunction of cilia in these regions results in an impaired myocardial proliferation, and finally to the occurrence of thinner ventricular walls and the development of VSDs in mice. In this context, HH signalling governs the outgrowth of the muscular part of the VS. However, the membranous part of the VS is indirectly affected since an impaired formation of the muscular VS impedes the development of the membranous septum [119]. Interestingly, HH signalling ensures the proper ciliary localization of PDGFRα in these cardiac cilia [119]. In combination with the fact that *Pdgfrα* mutant mouse embryos exhibit VSDs [120,121], it is likely that intracardiac HH signalling regulates VS formation via controlling PDGFRα signalling [119]. To sum up, the formation of the VS is regulated via HH signalling in the pharyngeal endoderm, at the left side of the node and in the ventricular walls. Any impairment of the HH signalling transduction cascade in these different areas and the associated different processes can lead to the occurrence of VSDs. Consequently, HH signalling plays an important role in proper VS genesis.

## 3. Is It Possible to Prevent the Development of VSDs by Targeting HH Signalling in Pregnancy?

Once a large VSD has been diagnosed in a newborn, it is difficult to imagine that a pharmacological treatment is able to close the defect. Consequently, maternal exposure to small molecules during pregnancy could be an option to prevent the development of VSDs. A good example for such a therapy is the preconceptional intake of folic acid to avoid the development of neural tube defects [122]. When considering that decreased HH signalling can result in the occurrence of VSDs, the restoration of disturbed HH might prevent the development of VSDs. In the case of reduced HH signalling, SMO agonists are promising candidates for therapeutic approaches [123]. Known SMO agonists are the benzothiophene SAG [124], the trisubstituted purine purmorphamine [125], oxysterols [126] and the quinolinone GSA-10 [127]. They control SMO activity via a direct interaction. However, the use of these small molecules for therapeutic purposes has to be extensively tested since hyperactivated HH signalling has teratogenic potential entailing several risks, like, for example, the formation of tumors in certain organs [128,129,130,131,132,133,134,135,136,137]. Furthermore, it was reported that a single injection of SAG in pregnant mice at E9.25 leads to pre-axial polydactyly in their embryos [138]. In this context, it cannot be excluded that SMO agonists have an effect on other signalling pathways that are involved in VS formation, like the canonical WNT pathway, the NOTCH pathway, or the TGF-β pathway. The general point of view is that HH agonists might influence other pathways indirectly via stimulating HH signalling. Since there are also cross-reactions of canonical WNT signalling, NOTCH signalling and TGF-β signalling with the repressor arm of HH signalling (e.g., GSK3-β) [139,140], it is even possible that HH agonists indirectly activate GLI2-R and GLI3-R. A recent report showed that SAG is not able to alter canonical Wnt signalling in murine F9 cells (teratocarcinoma stem cells [141]) [142], but, to our knowledge, such studies were never performed in cardiac cells. Accordingly, it will be a difficult task in future to stimulate HH signalling to an adequate degree at the convenient time.

Patients suffering from Down syndrome often develop VSDs [143,144,145,146]. As mentioned before, a study showed a relation between a decreased expression of the HH target gene GLI1 and the appearance of VSDs in Down syndrome patients [91], indicating that reduced HH signalling provokes VSDs in these patients. This hypothesis is supported by the fact that there are significant similarities between the heart phenotypes that are observed in HH signalling mutant mice and those seen in Down syndrome mouse models [147]. In regard to the development of VSDs, it is remarkable that a hypoplastic dorsal mesenchymal protrusion (a tissue derived from the second heart field) was found in Down syndrome patients and mouse models [148,149,150]. Under the assumption that reduced HH signalling causes several defects in Down syndrome patients, a mouse model of Down syndrome was treated with SAG. Injection of SAG into newborn pups corrected cerebellar dysmorphology [151] and hippocampal function [152], but cerebellar function is not restored completely [153]. Until now, studies that describe the effect of SAG treatment on the frequency of VSDs in Down syndrome mice were not reported. Since VS formation is already finished at birth, it would be necessary to treat pregnant Down syndrome mice with SAG and to analyse the VS phenotype of their progeny. Potentially, studies in Down syndrome mouse models might provide the opportunity to test HH signalling agonists as promising candidates to realize the development of a pharmacological therapy to prevent the occurrence of VSDs.

## Figures and Tables

**Table 1:** Heart phenotypes of humans and mice mutant for genes encoding Hedgehog signalling components.

Gene Symbol	Cardiac Phenotype	Literature
*Shh*	AVSDs Arch artery and outflow tract patterning defects Abnormal development of migratory neural crest cells	[71]
*Lrp2*	VSDs Aortic arch defects Persistent truncus arteriosus	[34]
*Sufu*	VSDs Coronary artery abnormalities Double outlet right ventricle/overriding aorta	[34]
*Bromi* (*Tbc1d32*)	AVSDs Congenital heart defects	[34]
*Ift25*	AVSDs VSDs Double outlet right ventricle/overriding aorta Common atrium Outflow tract malalignment defects	[57,58]
*Ift27*	AVSDs VSDs Double outlet right ventricle Common atrium Hypoplasia of the pulmonary trunc Pulmonary artery defects Aortic arch anomalities	[57,58]
*EVC1*	AVSDs Common atrium Persistent superior left vena cava	[86,87]
*EVC2*	AVSDs Common atrium Persistent superior left vena cava	[86,87]
*Kif7*	AVSDs Double outlet right ventricle/overriding aorta Pulmonary artery hypoplasia Interrupted aortic arch	[34,73]
*Fuz*	AVSDs Dual inferior vena cava Multiple major aortopulmonary collateral arteries pulmonary valve atresia right aortic arch	[34,73]
*Gsk3-β*	VSDs Atrioventricular canal defect Double outlet right ventricle	[72]
